# White Matter Correlates of Hostility and Aggression in the Visuospatial Function Network in Patients With Schizophrenia

**DOI:** 10.3389/fpsyt.2021.734488

**Published:** 2021-10-07

**Authors:** Iseul An, Tai Kiu Choi, Minji Bang, Sang-Hyuk Lee

**Affiliations:** ^1^Department of Psychiatry, CHA Bundang Medical Center, CHA University School of Medicine, Seongnam, South Korea; ^2^Clinical Counseling Psychology Graduate School, CHA University, Seongnam, South Korea; ^3^Department of Clinical Pharmacology and Therapeutics, CHA Bundang Medical Center, CHA University School of Medicine, Seongnam, South Korea

**Keywords:** schizophrenia, aggression, visuospatial, brain imaging (MRI), white matter connectivity

## Abstract

**Background:** Violent acts in patients with schizophrenia are often associated with their hostility and aggression levels. Poor visuospatial processing has been suggested as a possible risk factor of violence in schizophrenia. However, studies investigating the relationship between hostility, aggression, and the visuospatial function have been lacking. Here, we aimed to investigate brain dysconnectivity associated with hostility and aggression in schizophrenia, particularly focusing on the visuospatial function network.

**Methods:** Eighty-eight participants with schizophrenia and 42 healthy controls were enrolled. The visuospatial function network regions of interest were analyzed using Tract-Based Spatial Statistics. The hostility item from the Positive and Negative Syndrome Scale (PANSS), aggressive, and agitated behavior item from the Scale for the Assessment of Positive Symptoms (SAPS), and the Rey Complex Figure Test (R-CFT) were measured.

**Results:** Among the participants with schizophrenia, the SAPS aggressive and agitated behavior scores were significantly correlated with fractional anisotropies (FAs) of the white matter regions in the splenium of the corpus callosum (CC), left posterior thalamic radiations (PTR), and left posterior corona radiata (PCR). Exploratory correlational analysis revealed significant negative correlations between FAs of the splenium of the CC and R-CFT copy and immediate recall scores. In addition, three regions including CC, PTR, and PCR that significantly correlated with the aggression scores showed significant correlations with the total PANSS scores.

**Conclusion:** Our main finding suggests that aggression of patients with schizophrenia may be associated with poor visuospatial ability and underlying white matter dysconnectivity. These may help enhance understanding aggression in patients with schizophrenia.

## Introduction

There have been increasing concerns about violence perpetrated by patients with schizophrenia (1–3). Although the evidence on the causality remains inconclusive, several large population-based studies have shown a significant association between violence and schizophrenia (4, 5). A major source of violent acts in patients with schizophrenia is often related to the levels of hostility and aggression, which lay a substantial burden upon their closest caregivers in daily living (6–8). Hostility refers to an enduring attitude of ill will and negative evaluation of others (9), and aggression is defined as any behavior directed toward another individual that is carried out with the proximate intent to cause harm (10). Both hostility and aggression could worsen clinical outcomes of patients with schizophrenia, including lengthening of hospitalization and increasing costs (11), stigmatization (12), and poor treatment adherence (6).

Several neuroimaging studies have suggested that hostility and aggression in patients with schizophrenia are related to dysfunction of certain brain networks (13). A structural brain magnetic resonance imaging (MRI) study revealed that patients with schizophrenia who had a history of violence showed significantly smaller gray matter (GM) volumes in the right inferior temporal area compared to those with no history of violence (14). In studies of chronic patients with schizophrenia or schizoaffective disorder, higher levels of aggression were found to be correlated with larger GM and white matter (WM) volumes in the orbitofrontal cortex (15) and larger caudate volumes (16). Functional brain MRI studies found that violent patients showed relative hyperactivity in the medial frontal and anterior cingulate regions during negative emotion processing, compared to non-violent patients (17, 18). Although the neurobiological pathology underlying hostility and aggression is still not clear (19, 20), it has been consistently found that structural and functional abnormalities in the fronto-temporo-limbic circuits are significantly associated with hostility and aggression in patients with schizophrenia.

Meanwhile, deficits in sensory-perceptual processing, consistently observed in patients with schizophrenia (21–23), have been considered an important precursor to violent behaviors (24). Aberrant processing of sensory stimuli, particularly of a visuospatial nature, could make them prone to develop biased emotional and behavioral responses (25–28). Given that the visual system is the most dominant source of sensory inputs from our environment, brain circuits recruited for the visuospatial function are expected to play a substantial role in hostility and aggression in patients with schizophrenia. The visuospatial function network, including the fronto-parieto-occipital cortices and thalamus, involves emotional processing of visual stimuli, motivated attention to the significance, and sensory distinctiveness of the stimuli (29–31). The WM regions in the fronto-parieto-occipital cortices and thalamus contain the posterior limb of the internal capsule, posterior corona radiata (PCR), posterior thalamic radiation (PTR), corpus callosum (CC) splenium, superior longitudinal fasciculus (SLF), sagittal stratum, and superior fronto-occipital fasciculus (SFOF) (32). These regions are composed of neuronal fibers interconnecting the thalamus, visual association area near the occipital lobe, and sensory motor cortex, which play an important role in behavioral decisions in response to external stimuli by integrating and transmitting the visual stimuli and influencing the autonomic emotional responses in the amygdala, hypothalamus, and midbrain (32–36). In a recent study of healthy individuals, high trait anger was associated with aberrant neural responses to unpleasant emotional stimuli in brain regions consisting of the visuospatial function network (31). However, whether and how hostility and aggression are related to abnormalities in the visuospatial function network have never been investigated in patients with schizophrenia.

The present study aimed to investigate the WM correlates of hostility and aggression in patients with schizophrenia, focusing on the visuospatial function network. Based on the notion of schizophrenia as a disorder of aberrant brain connectivity (37), hostility and aggression in schizophrenia would be better understood by exploring miscommunication between several brain regions rather than specific abnormalities in a single distinct area. Diffusion tensor imaging (DTI) was used to assess WM connectivity at a microscopic scale by quantifying the degree of water diffusion along neuronal fibers (38). We hypothesized that (1) structural connectivity within WM tracts consisting of the visuospatial function network would be associated with the levels of hostility and aggression in patients with schizophrenia and that (2) WM aberrations associated with hostility and aggression would be correlated with the poorer visuospatial ability and more severe clinical symptoms.

## Materials and Methods

### Participants

Participants with schizophrenia were recruited from the Department of Psychiatry, CHA Bundang Medical Center (Seongnam, Republic of Korea), from January 2011 to September 2019. All participants were first diagnosed as having schizophrenia, according to the criteria from the Diagnostic and Statistical Manual of Mental Disorders, Fourth Edition, Text Revision (DSM-IV-TR). The diagnostic interview was conducted by experienced psychiatrists using the Structured Clinical Interview for DSM-IV-TR Axis I Disorders (39). Participants with a current or past history of mood disorders, alcohol and substance use disorders, intellectual disability, head trauma with loss of consciousness, or neurological diseases were excluded. We also excluded participants who were left-handed and contraindicated with MRI. Handedness was assessed using the Edinburgh Handedness Inventory (40). Overall, 88 participants with schizophrenia were finally included in the present study.

Healthy controls (HCs) were recruited using online and print advertisements from the local community between May 2011 and September 2019. They were excluded if they had a current or past history of psychiatric disorders, neurological diseases, head trauma causing loss of consciousness, or a history of any major psychiatric disorders among their first-degree relatives. HCs were matched with participants in the schizophrenia group with respect to age, sex, and handedness by a blinded investigator independent of the present study, and 42 HCs were finally included.

All study procedures were approved by the Institutional Review Board of CHA Bundang Medical Center, in accordance with the Declaration of Helsinki and principles of Good Clinical Practice. After providing an explanation and written description of the study procedures, written informed consent was obtained.

### Clinical Assessments

Patients were assessed for hostility, aggression, and visuospatial function using the Positive and Negative Syndrome Scale (PANSS; P7 hostility) scores (41), Scale for the Assessment of Positive Symptoms (SAPS; aggressive and agitated behavior) scores (42), and Rey Complex Figure Test (R-CFT) scores, respectively. The overall severity of clinical symptoms at baseline was measured using the PANSS total scores. The R-CFT is a neuropsychological assessment method in which examinees are asked to reproduce a complicated line drawing, first by copying it freehand (recognition) and then by drawing from memory (recall). The R-CFT includes the component of visuospatial function of copy, immediate recall, and delay recall (43, 44).

### MRI Data Acquisition

All MRI scans were obtained at CHA Bundang Medical Center at CHA University on the same 3.0 T GE Signa HDxt scanner (GE Healthcare, Milwaukee, WI, USA) equipped with an eight-channel phased array head coil. Diffusion-weighted images (DWIs) were acquired using an echo-planar imaging (EPI) sequence, with the following parameters: TR, 17,000 ms; TE, 107.5 ms; FOV, 24 cm; matrix, 144 × 144; slice thickness, 1.7 mm; and voxel size, 1.67 × 1.67 × 1.7 mm^3^. A double-echo option was used to reduce eddy current-related distortions. To reduce the impact of EPI spatial distortions, an eight-channel coil and Array of Spatial Sensitivity Encoding Techniques (ASSET; GE Healthcare) with a sensitivity encoding speed-up factor of 2 was used. Seventy axial slices parallel to the anterior commissure-posterior commissure line were acquired in 51 directions with *b* = 900 s/mm^2^. Eight baseline scans with *b* = 0 s/mm^2^ were also acquired. Diffusion tensor images were calculated from the DWIs using the least-squares method with Functional MRI of the Brain (FMRIB) Software Library (FSL; version 5.0; Oxford, UK; https://fsl.fmrib.ox.ac.uk/fsl/).

### Diffusion Tensor Imaging Analysis

DTI data were analyzed using FMRIB Diffusion Toolbox and Tract-Based Spatial Statistics (TBSS) (45), implemented in the FSL. All images were checked by visual inspection for major artifacts, such as insufficient image acquisition, geometric distortions, and signal dropouts. No images were discarded as outliers or were removed from the data analysis. DTI preprocessing was performed according to the standard procedure. Eddy current- and motion-related distortions were corrected, and the b-vectors were rotated accordingly (46). Skull stripping was performed using the Brain Extraction Tool. Fractional anisotropy (FA) images were obtained by fitting a tensor model to the raw diffusion data (47). Subsequently, the FA data of all subjects were aligned into the standard space (Montreal Neurologic Institute 152 standard) using FMRIB's Nonlinear Image Registration Tool. All transformed FA images were combined and applied to the original FA map, producing a standard-space version FA map. Furthermore, all transformed FA images were averaged to make a mean FA image, which was then thinned (skeletonized) to yield a mean FA skeleton, taking only the centers of WM tracts. The threshold of FA > 0.2 (TBSS default) was applied to the skeleton to involve only major fiber bundles. Non-FA images (axial diffusivity [AD], radial diffusivity [RD], and mean diffusivity [MD]) were prepared in a similar way according to the non-FA pipeline in the FSL.

**Figure 1:**
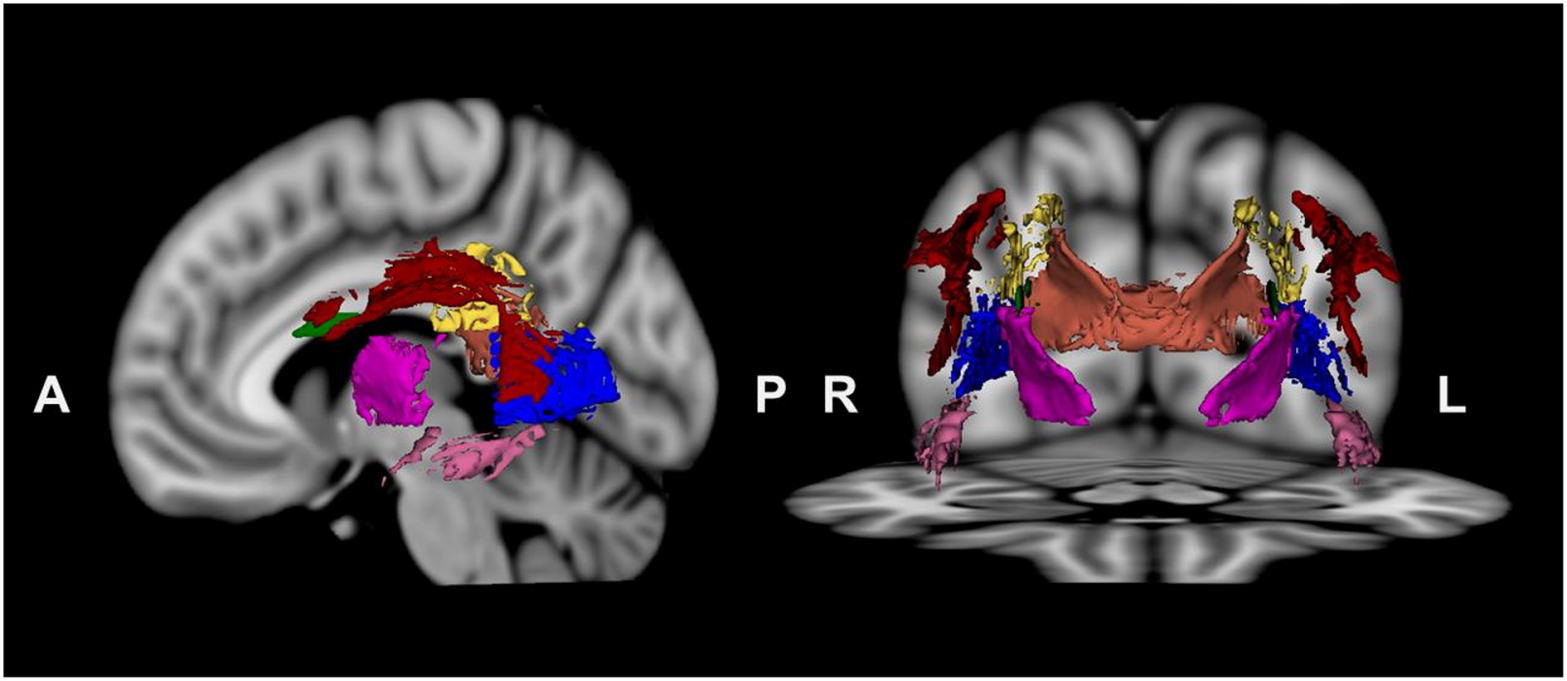
Visuospatial function network regions, including the superior longitudinal fasciculus (red), posterior thalamic radiation (blue), posterior corona radiata (yellow), splenium of the corpus callosum (orange), posterior limb of the internal capsule (dark pink), sagittal stratum (pink), and superior fronto-occipital fasciculus (green), were chosen.

The visuospatial function network regions of interest (ROIs) were selected according to the Johns Hopkins University DTI-based WM atlas (48) and extracted using 3D Slicer, version 4.3.1 (49). Since the visuospatial function network includes the fronto-parieto-occipital cortex and thalamus (29–31), we selected the following WM ROIs: the SLF, PTR, PCR, CC splenium, posterior limb of the internal capsule, sagittal stratum, and SFOF (Figure 1). The ROI mask was created by multiplying the mean FA skeleton mask by the regional mask of the WM underlying the visuospatial function network. Voxel-wise statistical analysis was performed within the ROI mask using permutation-based nonparametric inference (computing 5,000 permutations). We used a voxel-wise *t*-test to compare the WM connectivity of the visuospatial function network between participants with schizophrenia and HCs. We controlled for sociodemographic differences as covariates. DTI data were evaluated using TBSS general linear model regression analysis with SAPS-aggression or PANSS-hostility scores as a factor for correlation analysis. Multiple comparisons were corrected using the threshold-free cluster enhancement (TFCE) method. Statistical significance was set at a TFCE-corrected value of *p* < 0.05.

### Statistical Analysis

Independent *t*- and chi-square tests were used to compare demographic and clinical characteristics between participants with schizophrenia and HCs. Correlation analysis was performed to examine the relationships between the R-CFT scores and DTI measures, extracted from clusters showing significant correlations with SAPS-aggression or PANSS-hostility scores in participants with schizophrenia. We further investigated the correlations between DTI measures and PANSS total scores in the same manner. We controlled for age, sex, years of education, intracranial volume, duration of illness, duration of untreated psychosis, presence of a family history of psychotic disorders, chlorpromazine equivalent doses, kinds of medication, and duration of drug therapy at the time of scanning as covariates. All statistical analyses were performed using SPSS Statistics software (version 26.0; IBM Corporation, Armonk, NY, USA). The statistical significance for the correlations was presented as uncorrected for multiple comparisons unless otherwise indicated.

## Results

### Sociodemographic and Clinical Characteristics

There were no significant differences between the two groups in terms of sex and age at the time of the brain MR scan except education level. All patients were taking antipsychotic medication at the time of the brain MR scan, including paliperidone, olanzapine, risperidone, amisulpride, clozapine, and aripiprazole. Antipsychotic medication doses were calculated as equivalent to chlorpromazine (50), which was 232.83 ± 237.75 mg per day. Most participants with schizophrenia (71 of 88; 80.7%) were first exposed to antipsychotic medication. The average duration of medication was relatively short, at 6.70 ± 5.85 days. Table 1 outlines the detailed sociodemographic and clinical characteristics of participants with schizophrenia and HCs (Table 1).

**Table 1:** Sociodemographic and clinical characteristics of participants with schizophrenia and healthy controls.

	**Schizophrenia (*n* = 88)**	**Healthy controls (*n* = 42)**	**Statistics**	***p-*value**
Sex (*n*, male/female)	27/61	18/24	*x^2^* = 1.862	0.172
Age at scan (years, mean ± SD)	35.60 ± 12.07	38.40 ± 9.97	*t* = −1.317	0.190
Education (years, mean ± SD)	13.44 ± 2.61	16.53 ± 3.06	*t* = −5.677	0.000
Intracranial volume (ml, mean ± SD)	1,575.90 ± 305.19	1,537.61 ± 124.41	*t* = 0.815	0.417
Duration of illness (months, mean ± SD)	32.70 ± 67.34			
Duration of untreated psychosis (months, mean ± SD)	13.86 ± 41.36			
Presence of family history (*n*, yes)	31			
Duration of antipsychotics at scan (days, mean ± SD)	6.70 ± 5.85			
Dose of antipsychotics at scan^*^ (mg, mean ± SD)	232.83 ± 237.75			
Types of antipsychotics				
Paliperidone (*n*)	26			
Olanzapine (*n*)	3			
Risperidone (*n*)	9			
Amisulpride (*n*)	12			
Clozapine (*n*)	2			
Aripiprazole (*n*)	1			
PANSS, total scores (mean ± SD)	116.10 ± 27.60			
Positive symptom (mean ± SD)	30.30 ± 7.01			
Negative symptom (mean ± SD)	27.81 ± 9.03			
General psychopathology (mean ± SD)	58.07 ± 14.34			
SAPS, total scores (mean ± SD)	73.20 ± 30.61			
SANS, total scores (mean ± SD)	60.42 ± 28.47			
Hostility item of the PANSS (mean ± SD)	3.64 ± 1.83			
Aggressive and agitated behavior item of the SAPS (mean ± SD)	2.88 ± 1.60			
R-CFT				
Copy (mean ± SD)	11.08 ± 5.06			
Immediate recall (mean ± SD)	7.75 ± 4.98			
Delayed recall (mean ± SD)	12.14 ± 22.15			

*PANSS, Positive and Negative Syndrome Scale; SAPS, Scale for the Assessment of Positive Symptoms; SANS, Scale for the Assessment of Negative Symptoms; R-CFT, Rey Complex Figure Test; SD, standard deviation*.

* *Doses of antipsychotics were converted to the equivalent of chlorpromazine*.

### WM Connectivity of the Visuospatial Function Network and Its Association With Hostility and Aggression in Patients With Schizophrenia

**Figure 2:**
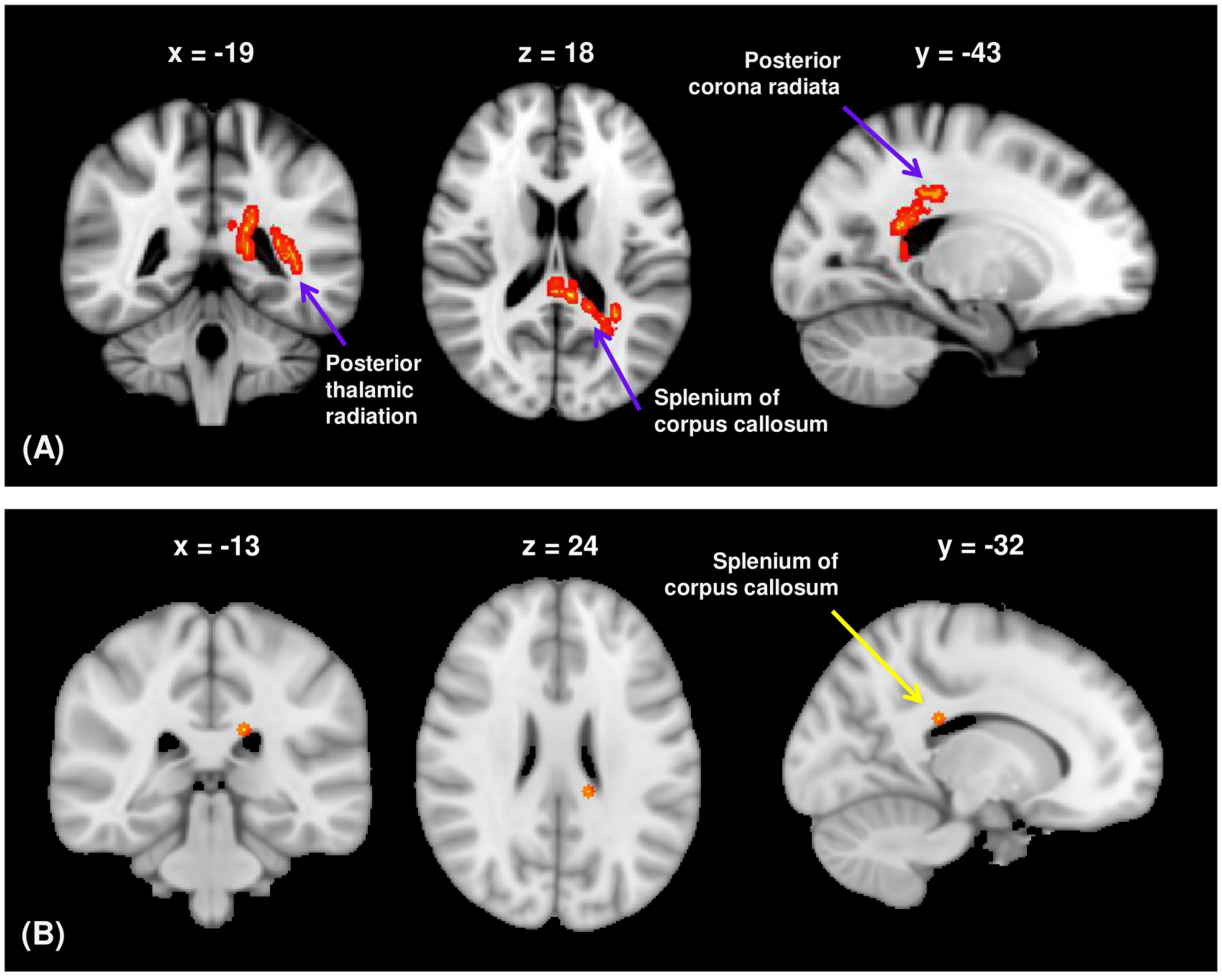
**(A)** The FA of the white matter regions showed significant positive correlations with the aggressive and agitated behavior scores from the SAPS in participants with schizophrenia (TFCE-corrected *p* < 0.05). **(B)** The RD of the splenium of corpus callosum showed significant negative correlations with the aggressive and agitated behavior scores from the SAPS in participants with schizophrenia (TFCE-corrected *p* < 0.05). FA, fractional anisotropy; RD, radial diffusivity; SAPS, Scale for Assessment of Positive Symptoms; TFCE, threshold-free cluster enhancement.

Among the visuospatial function WM networks, the FA values of the CC splenium, PTR, right of the PCR, right of the posterior limb of the internal capsule, and right of the SFOF were significantly lower in participants with schizophrenia than in HCs after controlling for years of education (TFCE-corrected *p* < 0.05; Supplementary Figure 1). SAPS aggressive and agitated behavior scores were significantly and positively correlated with FA values of the CC splenium, left PTR, and left PCR in the visuospatial function network (TFCE-corrected *p* < 0.05; Figure 2A). There was a significant negative correlation between SAPS aggressive and agitated behavior scores and RD values of the CC splenium in participants with schizophrenia (TFCE-corrected *p* < 0.05; Figure 2B). AD and MD values were not significantly correlated with SAPS-aggression scores. The mean FA values extracted from the significant clusters of the CC splenium, left PTR, and left PCR remained significantly correlated with SAPS aggressive and agitated behavior scores after controlling for age, sex, years of education, intracranial volume, duration of illness, duration of untreated psychosis, presence of a family history of psychotic disorders, chlorpromazine equivalent doses, kinds of medication, and duration of drug therapy at the time of scanning as covariates (CC splenium: *r* = 0.484, *p* < 0.001; left PTR: *r* = 0.383, *p* = 0.001; left PCR: *r* = 0.488, *p* < 0.001). The mean RD values extracted from the significant clusters of the CC splenium were not significantly correlated with SAPS aggressive and agitated behavior scores after controlling for the above variables (*r* = 0.205, *p* = 0.082). The PANSS hostility scores showed no significant correlations with any DTI measures.

### Exploratory Correlational Analysis Between the FA Values of the Visuospatial Function WM Regions Significantly Correlated With Aggression and Visuospatial Function in Schizophrenia

**Figure 3:**
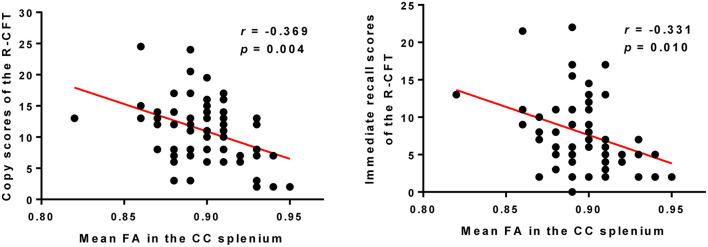
Higher FA in the CC splenium was correlated with poorer copy and immediate recall scores of the R-CFT in participants with schizophrenia. R-CFT, Rey Complex Figure Test; FA, fractional anisotropy; CC, corpus callosum.

The mean FA values extracted from the cluster of the CC splenium, which showed a significant correlation with SAPS aggressive and agitated behavior scores, showed negative correlations with the R-CFT copy and immediate recall scores (Figure 3). The R-CFT delayed recall scores were not significantly correlated with the mean FA values of the CC splenium. The mean FA values extracted from the clusters of the left PTR and PCR were not significantly correlated with R-CFT scores. The region's mean RD values extracted from the CC splenium showed a significant correlation with SAPS aggressive and agitated behavior scores, but not with R-CFT scores.

### Exploratory Correlational Analysis Between the FA Values of the Visuospatial Function WM Regions Significantly Correlated With Aggression and Symptom Severity Measures in Schizophrenia

**Figure 4:**
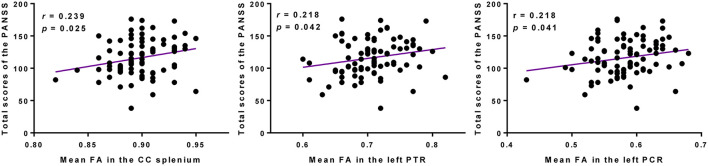
Higher FAs in the CC splenium, left PTR, and left PCR were correlated with more severe psychopathology symptoms in participants with schizophrenia. PANSS, Positive and Negative Syndrome Scale; FA, fractional anisotropy; CC, corpus callosum; PTR, posterior thalamic radiation; PCR, posterior corona radiata.

The mean FA values of WM regions that were significantly correlated with the SAPS aggressive and agitated behavior scores showed significant positive correlations with the PANSS total scores. The mean FA values of the CC splenium, left PTR, and left PCR showed significant positive correlations with the PANSS total scores (Figure 4). However, the mean RD values extracted from the CC splenium did not correlate with the PANSS total scores.

## Discussion

To the best of our knowledge, this is the first study to investigate the WM correlates of hostility and aggression within the visuospatial function network in patients with schizophrenia. Increased FA values of the CC splenium, left PTR, and left PCR and decreased RD values extracted from the CC splenium were associated with higher levels of aggression in the schizophrenia group. Furthermore, FAs in these regions showed positive correlations with the severity of clinical symptoms measured using the PANSS. Our findings suggest that dysconnectivity in WM tracts consisting of the visuospatial function network may underlie aggression in patients with schizophrenia.

Compared to HCs, participants with schizophrenia had lower FA values of the WM tracts in the CC splenium, PTR, right of the PCR, right of the posterior limb of the internal capsule, and right of the SFOF. This result is grossly in line with that of a previous ENIGMA study with large-size schizophrenia samples, which found decreased FAs in WM regions consisting of the visuospatial function network, including the SLF, PTR, and SFOF (51). However, the FA values of the right of the PTR, right of the PCR, right of the posterior limb of the internal capsule, and right of the SFOF were not associated with hostility and aggression in participants with schizophrenia. Instead, FAs in the CC splenium, left PTR, and left PCR regions were positively correlated with not only higher aggression but also more severe clinical symptoms in our participants. Although the reason for these nonoverlapping findings cannot be established here, it may be assumed that differences in WM connectivity found in patients with schizophrenia are not necessarily associated with aggressive behaviors. Instead, WM connectivity associated with aggression may contribute to psychotic exacerbation in schizophrenia (52). Since aggression and violence significantly contribute to stigmatization and rejection of mentally ill people from their social communities (53), understanding the neurobiological origin of aggression in patients with schizophrenia is crucial to reduce biases against them.

At first glance, it seems counterintuitive that higher FA values were associated with higher aggression, poorer visuospatial performance, and more severe clinical symptoms. However, higher FA values do not always suggest enhanced brain function (54–56). In patients with schizophrenia, excessive excitation in the WM pathway due to gamma-aminobutyric acid (GABA) system dysfunction and hyperdopaminergic activity modulates synaptic maps, leading to deficient axonal pruning (57). Deficient axonal pruning may induce redundant networks and reduce the efficiency of information processing, as reflected by increased FA values in patients with schizophrenia (58). Reduced efficiency in information processing can lead to lower cognitive performance, deficits in behavioral regulation, and worsening symptoms along with difficulties in filtering sensory inputs and distorted perception (59–61). Therefore, we speculated that the aberrant WM connectivity due to the overexcited neuronal activity, which is shown as high FA and low RD values, would be associated with brain malfunctioning, including aggression, poor visuospatial performance, and severe clinical symptoms (62).

The CC is the most extensive WM tract that provides reciprocal signal transfer between the left and right hemisphere. The CC supports the information processing of input and output signals to facilitate coordination in thoughts and behaviors (63). Recent evidence suggests that interhemispheric inhibition mediated by the CC is related to anger and aggression (64). Increased left-to-right transcallosal inhibition was associated with a stronger attentional bias for angry faces (65). Although the role of CC in aggression is unclear, we found increased FA and decreased RD values of the CC splenium showed significant association with the levels of aggression in participants with schizophrenia. FA is a sensitive measure of water diffusion along WM tracts, determined by the structural features of the myelin sheath, axonal membrane, and neurofibrils, while RD is associated with myelin compactness. Although we cannot determine the neurobiological meaning of DTI measures, our current findings suggest that structural changes in the CC splenium related to the increased risk of aggression may partly be related to excessive myelination in patients with schizophrenia.

We also observed a negative correlation between FAs in the CC splenium and visuospatial ability measured using the R-CFT. The posterior part of the CC, including the splenium, is known to play an important role in the integration of perception and action within a subcortico-cortical network, promoting a unified experience between the two hemispheres (66). Successful copying of the complex figure reflects sufficient capacity for visuospatial perception and visual-motor function (67). The immediate recall condition is used to assess encoding and short-term retention of visual information, and its performance is strongly predicted by the figure copy scores (43). In this regard, our findings suggest that dysconnectivity of the CC splenium that was related to visuospatial perception and short-term visual memory may contribute to the increased risk for aggression in patients with schizophrenia.

The PTR connects the thalamus with the posterior visual brain areas. The thalamus is involved in processing and integrating sensory information that enters the brain (68). Visuospatial information, initially processed in the thalamus, is transferred to the parieto-occipital cortex through the PTR (69). In this study, we found that higher aggression was correlated with increased FA in the left PTR in schizophrenia. Given that deficits in early stages of sensory and perceptual processing lead to impaired higher-order cognitive functioning in patients with schizophrenia, our finding suggests that excessive thalamo-visual connections may affect abnormal responses in the association cortices, causing aggressive behaviors by the process of disposing immediately without high-level judgment (27, 70).

The corona radiata, a tract that carries ascending and descending information from the cerebral cortex, is related to different elements of cognitive control, including visuospatial memory (71, 72). In this study, the GM area near the left PCR that was significantly correlated with aggression was the inferior parietal lobule (IPL). This finding is consistent with that of a previous study that showed an increased activation in the left-lateralized IPL on exposure to unpleasant images in individuals with high trait anger (31). The IPL is concerned with multiple aspects of sensorimotor integration (73). Taken together, it may be assumed that increased connectivity in the WM regions of the visuospatial function network causes misinterpretation of neutral stimuli, sensitivity to minor stimuli, or induction of negative emotions to aggressive behaviors in patients with schizophrenia. However, it is unclear how the “relatively” increased connectivity in these regions is related to heightened aggression in schizophrenia. There might be possible explanations for these findings. First, disruption of the inhibitory GABA-ergic system in these regions might lead to overactivity in the WM regions (74, 75). Second, increased WM connectivity within visuospatial function network could be related to the hyperdopaminergic status in schizophrenia, which may cause excessive neuronal activity in the downward WM pathway. In addition, hostility did not significantly correlate with the mean FA values of the visuospatial function-related WM regions in participants with schizophrenia. Hostility is commonly defined as general dislike and negativity toward others over a range of situations. In contrast, aggression is defined as a willingness to inflict harm, regardless of whether this is behaviorally expressed and physical harm is sustained (76). Our findings may indicate that aggression related to a motive to inflict harm reflects a pathophysiological process different from hostility related to attitudes and negative cognitive bias. A previous study has shown that biased negative attitudes toward other group members are associated with increased activation in the lateral orbitofrontal cortex, while positive attitudes toward affiliated group members are related to activation in the medial orbitofrontal cortex (77, 78). According to the defense motivation system (79, 80), aggression may be more likely influenced by interactions between the thalamus, amygdala, and hypothalamic area, which mediate the autonomic emotional response based on sensory input processing.

There are some limitations to be considered in this study. First, hostility and aggression we measured may reflect state rather than trait characteristics of patients with schizophrenia. Although it takes a long enough period of time for certain factors to elicit structural changes in the brain, WM tracts are known to possess relatively higher neuroplastic properties than GM regions do. Future longitudinal studies will be required to figure out the causal relationship between state and trait aggression and neurobiological changes in the visuospatial function network. Second, antipsychotic medications might affect WM integrity, although there was a relatively short time when participants underwent MR scans (6.70 ± 5.85). Although most participants with schizophrenia (80.7%) were first exposed to antipsychotic medication in this study, future studies are needed to examine drug-naïve patients. Third, a crossing fiber problem may have influenced the results. The tensor model represents an independent, dominant direction; thus, the estimated orientation for voxels with a complex fiber structure may be ambiguous or misleading.

In conclusion, we found that increased WM integrity in the CC splenium, left PTR, and left PCR was correlated with higher levels of aggression in schizophrenia. Furthermore, a negative correlation was observed between FA in the CC splenium and visuospatial ability. Our main finding suggests that WM abnormality-related aggression is not only found in the well-known fronto-temporo-limbic area but also in the visuospatial function network and that aggression of patients with schizophrenia may be associated with poor visuospatial ability and underlying WM dysconnectivity.

## Data Availability Statement

The data supporting the findings of this study are not publicly available due to ethical restrictions for protecting participants' confidentiality and privacy but are accessible from the corresponding author on reasonable request with the approval of the Institutional Review Board of CHA Bundang Medical Center. Requests to access the datasets should be directed to Sang-Hyuk Lee (drshlee@cha.ac.kr).

## Ethics Statement

The studies involving human participants were reviewed and approved by the Institutional Review Board of CHA Bundang Medical Center. The patients/participants provided their written informed consent to participate in this study.

## Author Contributions

S-HL designed the study. IA, TC, and MB managed the participant recruitment and data acquisition and compiled the database. IA and MB conducted the data preprocessing and statistical analysis. S-HL and IA implemented the literature reviews and interpretation of data. IA wrote the first draft of the manuscript. S-HL, TC, and MB provided the critical revision of the manuscript. All authors contributed to and approved the final manuscript.

## Funding

This research was supported by the Basic Science Research Program through the National Research Foundation funded by Ministry of Science and ICT, Republic of Korea (Grand no. NRF-2019M3C7A1032262 to S-HL) and (Grand no. 2021R1C1C1012901 to MB).

## Conflict of Interest

The authors declare that the research was conducted in the absence of any commercial or financial relationships that could be construed as a potential conflict of interest.

## Publisher's Note

All claims expressed in this article are solely those of the authors and do not necessarily represent those of their affiliated organizations, or those of the publisher, the editors and the reviewers. Any product that may be evaluated in this article, or claim that may be made by its manufacturer, is not guaranteed or endorsed by the publisher.
