# Supplementary material for: White Matter Correlates of Hostility and Aggression in the Visuospatial Function Network in Patients With Schizophrenia
Source: Front Psychiatry. 2021 Oct 7;12:734488. doi: 10.3389/fpsyt.2021.734488 (PMC8529184; doi:10.3389/fpsyt.2021.734488)
Supplement: Supplementary file 1 [file Data_Sheet_1.docx]

Supplementary Material

# Supplementary Figure


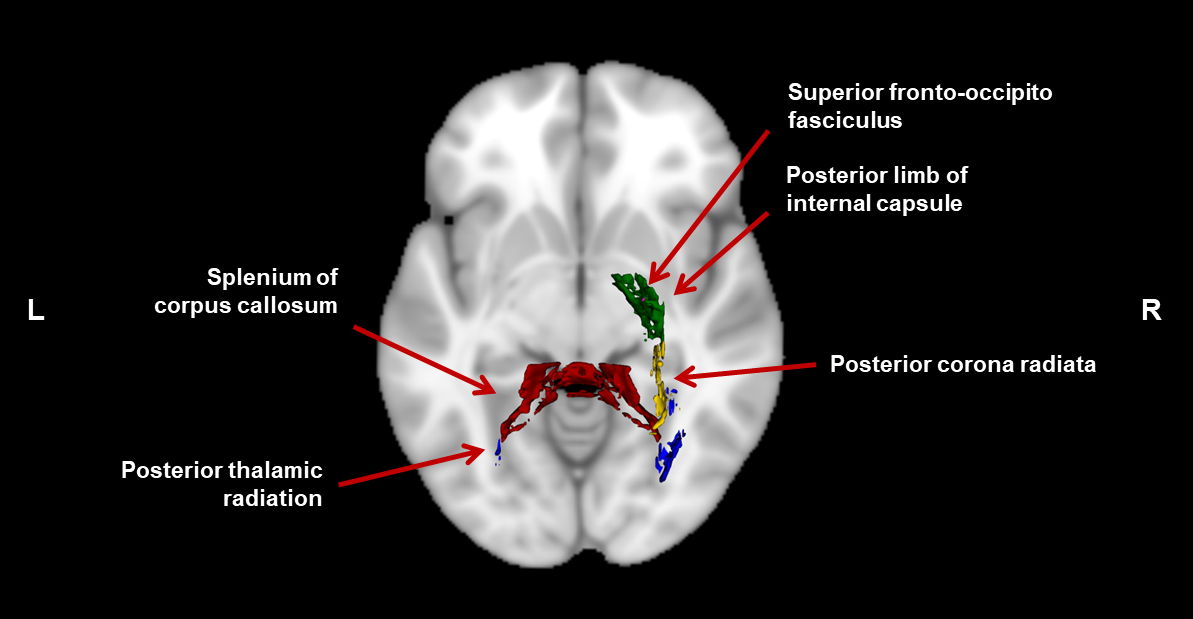


**Supplementary Figure 1.** The white matter regions, including the posterior limb of internal capsule (green), the superior fronto-occipito fasciculus (pink), the posterior corona radiata (yellow), the splenium of corpus callosum (red), and posterior thalamic radiation (blue), showed significant FA decreases in participants with schizophrenia compared to healthy controls after controlling years of education (TFCE-corrected *p* < 0.05).

Abbreviations: FA, fractional anisotropy; TFCE, threshold-free cluster enhancement.
